# Supplementary material for: ACTL6A protects gastric cancer cells against ferroptosis through induction of glutathione synthesis
Source: Nat Commun. 2023 Jul 13;14:4193. doi: 10.1038/s41467-023-39901-8 (PMC10345109; doi:10.1038/s41467-023-39901-8)
Supplement: Supplementary file 1 — Supplementary Information [file 41467_2023_39901_MOESM1_ESM.pdf]

ACTL6A Protects Gastric Cancer Cells against Ferroptosis through Induction of Glutathione Synthesis

Supplementary information

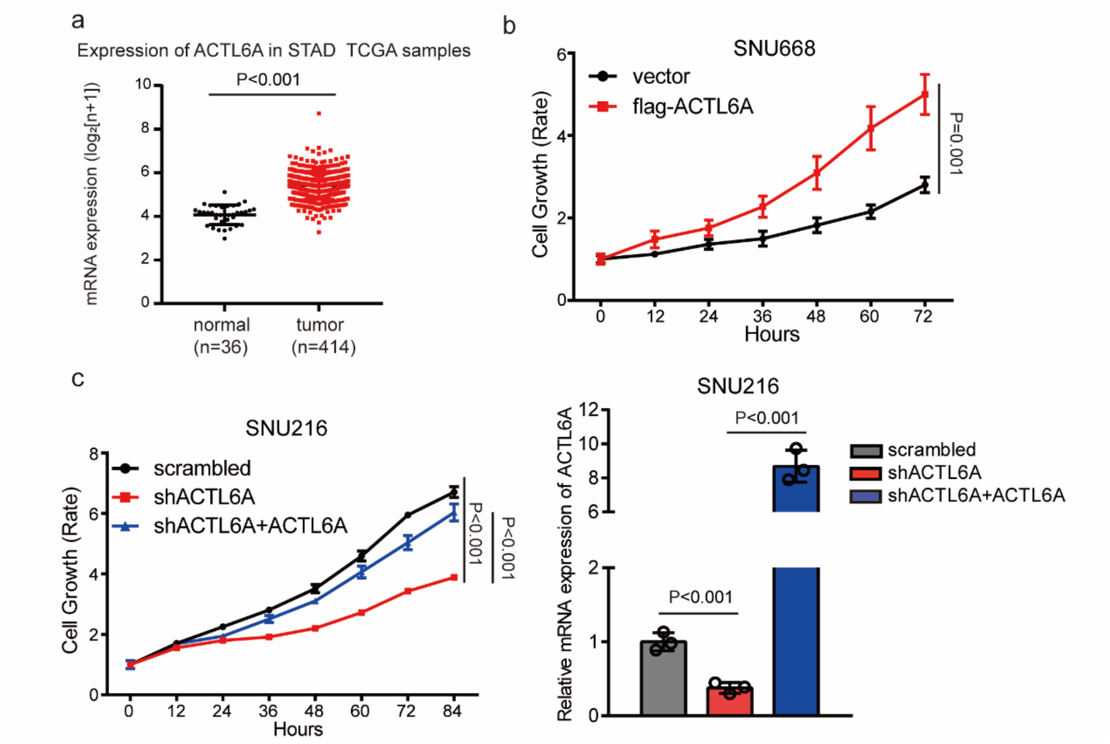

Supplementary figure 1 ACTL6A is overexpressed in GC and promotes GC progress. a. ACTL6A expression levels (log_2[n+1]) in gastric cancer and normal tissue from TCGA. The data are presented as the means ± SD. **b**. Relative cell growth rate of SNU668 cells transfected with flag-ACTL6A or pcDNA3.1 vector. The data are presented as the means ± SEM, n=3 biologically independent experiments. **c**. Relative cell growth rate of SNU216 cells treated with ACTL6A shRNA or flag-ACTL6A rescued. The data are presented as the means ± SEM, n=3 biologically independent experiments. **d**. mRNA expression levels of ACTL6A in cells from (**c**). Data are presented as the means ± SD, n=3 biologically independent experiments. *P* values were determined by unpaired two-tailed T test for panels **a, d**, and two-way ANOVA followed by Tukey test for panels **b, c**.

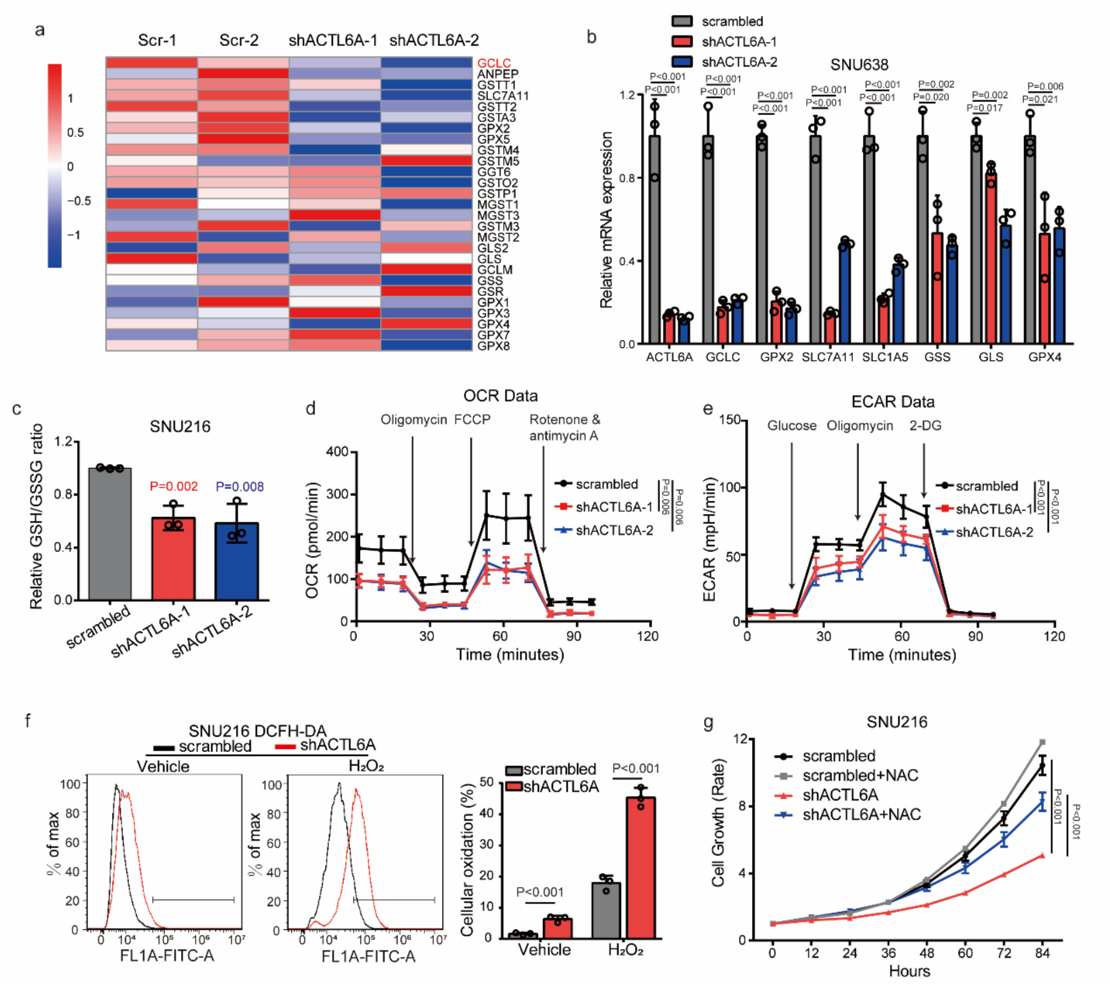

Supplementary figure 2 ACTL6A reprograms GSH metabolism to maintain GC malignant progression. a. Heatmap of GSH metabolism related genes in RNA array results. The color scale indicates fold change of log2 signal intensities of indicated genes. **b**. Gene abundance of the GSH metabolism pathway after knockdown of ACTL6A using shRNA. The data are presented as the means ± SD, n=3 biologically independent experiments. **c**. Measurement of relative GSH/GSSG ratio in SNU216 cells treated with ACTL6A shRNA and scrambled shRNA. The data are presented as the means ± SD, n=3 biologically independent experiments. **d-e**. Measurements of OCR (**d**) and ECAR (**e**) in SNU638 cells treated with ACTL6A shRNA or scrambled shRNA. The data are presented as the means ± SD, n=3 biologically independent experiments. **f**. DCFH-DA fluorescence intensity measured by flow cytometry, SNU216 cells are treated with ACTL6A shRNA and scrambled

shRNA and cultured with or without 50μM H_2O_2 for 24 hours. The data are presented as the means ± SD, n=3 biologically independent experiments. **g**. Relative cell growth rate of SNU216 cells treated with ACTL6A shRNA or scrambled shRNA and cultured with or without 100μM NAC for the indicated time points. The data are presented as the means ± SEM, n=3 biologically independent experiments. *P* values were determined by unpaired two-tailed T test for panels **b, c, f**, and two-way ANOVA followed by Tukey test for panels **d, e, g**.

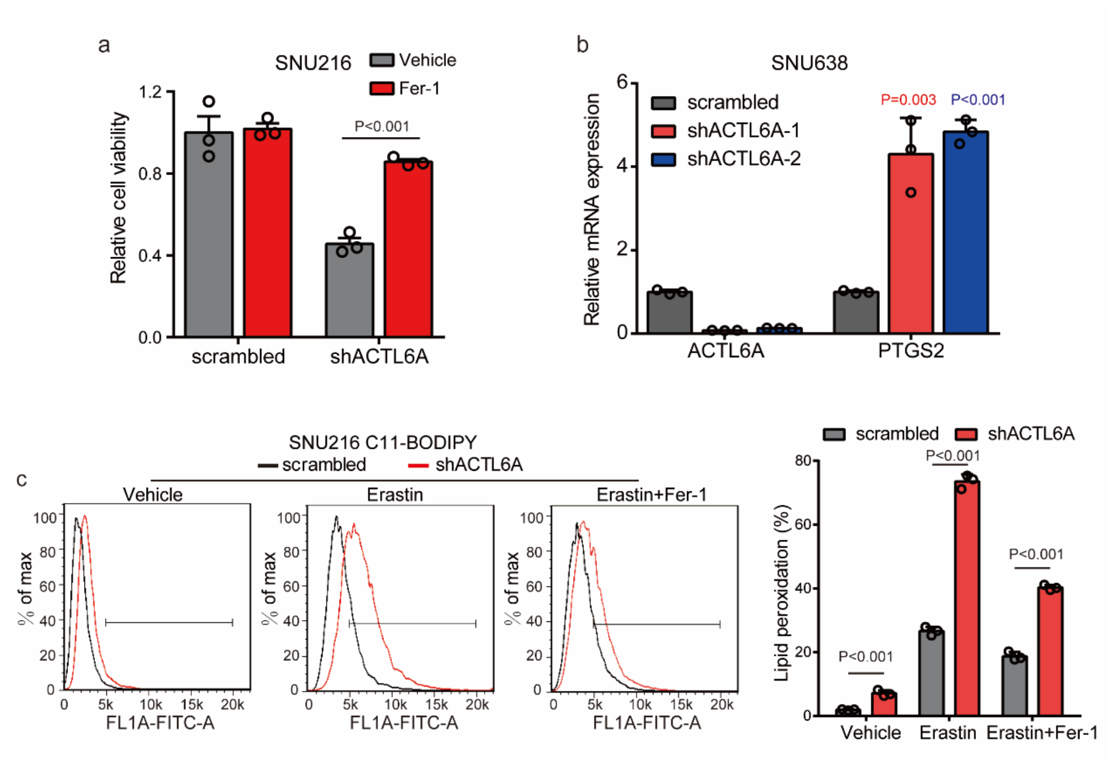

Supplementary figure 3 ACTL6A inhibits ferroptotic cell death. a. Viability of SNU216 cells treated with ACTL6A shRNA or scrambled shRNA after 24 hours cultured with or without 1μM Fer-1. The data are presented as the means ± SD, n=3 biologically independent experiments. **b**. mRNA expression levels of ACTL6A and PTGS2 in SNU638 cells treated with ACTL6A shRNA or scrambled shRNA. Data are presented as the means ± SD, n=3 biologically

independent experiments. **c**. C11-BODIPY fluorescence measured by flow cytometry of SNU216 cells treated with ACTL6A shRNA or scrambled shRNA and cultured with either erastin (10μM), Fer-1 (1μM) or both for 24 hours. The percentages of lipid peroxidation are presented as the means ± SD, n=3 biologically independent experiments. *P* values were determined by unpaired two-tailed T test for panels **a-c**.

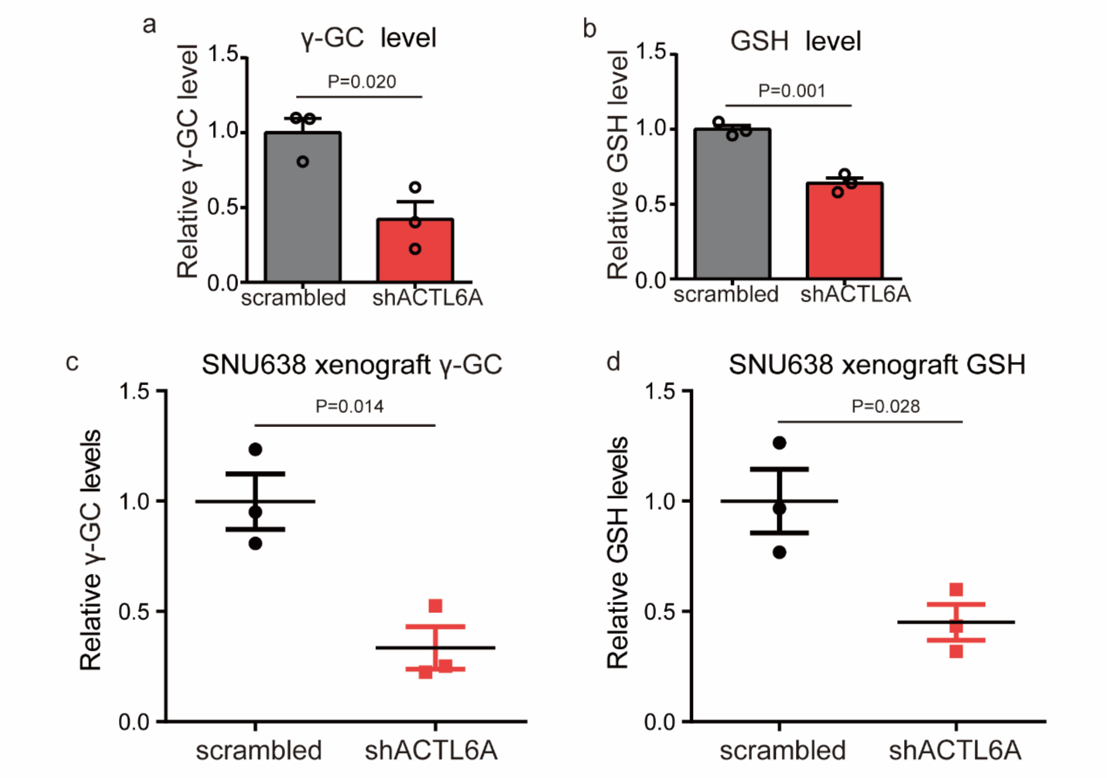

Supplementary figure 4 ACTL6A impacts GSH de novo synthesis mainly by upregulating γ-glutamyl-cysteine synthesis. a-b. Intracellular pool levels of γ-GC (**a**) and GSH (**b**) in scrambled and ACTL6A-KD SNU638 cells. The data are presented as the means ± SD. n=3 biologically independent experiments. **c-d**. Relative γ-GC (**c**) and GSH (**d**) in xenograft tissues derived from ACTL6A-knockdown or scrambled SNU638 cells in mice determined by LC–MS. The data are presented as the means ± SD. n=3 for each group. *P* values were determined by unpaired two-tailed T test for panels **a-d**.

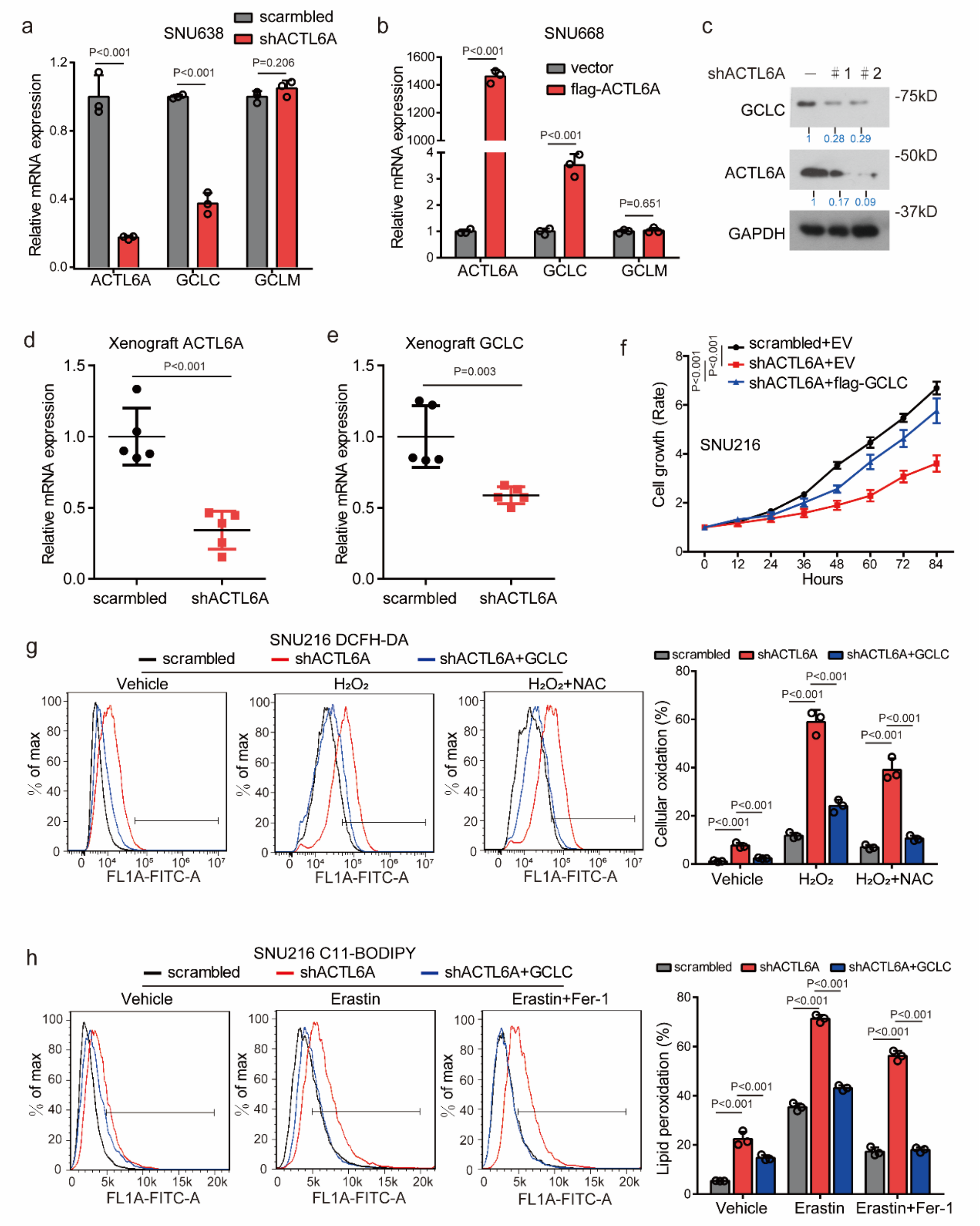

Supplementary figure 5 ACTL6A inhibits GC cell ferroptosis via regulating GCLC. a. mRNA expression levels of ACTL6A, GCLC and GCLM in SNU638 cells treated with ACTL6A shRNA or scrambled shRNA. Data are presented as the means ± SD, n=3 for each group. **b**. mRNA expression levels of ACTL6A, GCLC and GCLM in SNU668 cells transfected with flag-ACTL6A or pcDNA3.1 vector. Data are presented as the means ± SD, n=3 biologically

independent experiments. **c**. Immunoblot analysis of the GCLC and ACTL6A protein levels in SNU638 cells treated with ACTL6A shRNA or scrambled shRNA. **d-e**. mRNA expression levels of ACTL6A (**d**) and GCLC (**e**) in xenograft tissues derived from ACTL6A-knockdown SNU638 cells in mice determined. Data are presented as the means ± SD, n=5 for each group. **f**. Relative cell growth rate of SNU216 cells treated with ACTL6A shRNA or scrambled shRNA, and transfected with flag-GCLC or pcDNA3.1 vector. The Data are presented as the means ± SEM, n=3 biologically independent experiments. **g-h**. Relative DCFH-DA (**g**) and C11-BODIPY (**h**) fluorescence measured by flow cytometry of SNU216 cells with indicated treatment. The data are presented as the means ± SD, n=3 biologically independent experiments. *P* values were determined by unpaired two-tailed T test for panels **a-b, d-e, g-h**, and two-way ANOVA followed by Tukey test for panel **f**.

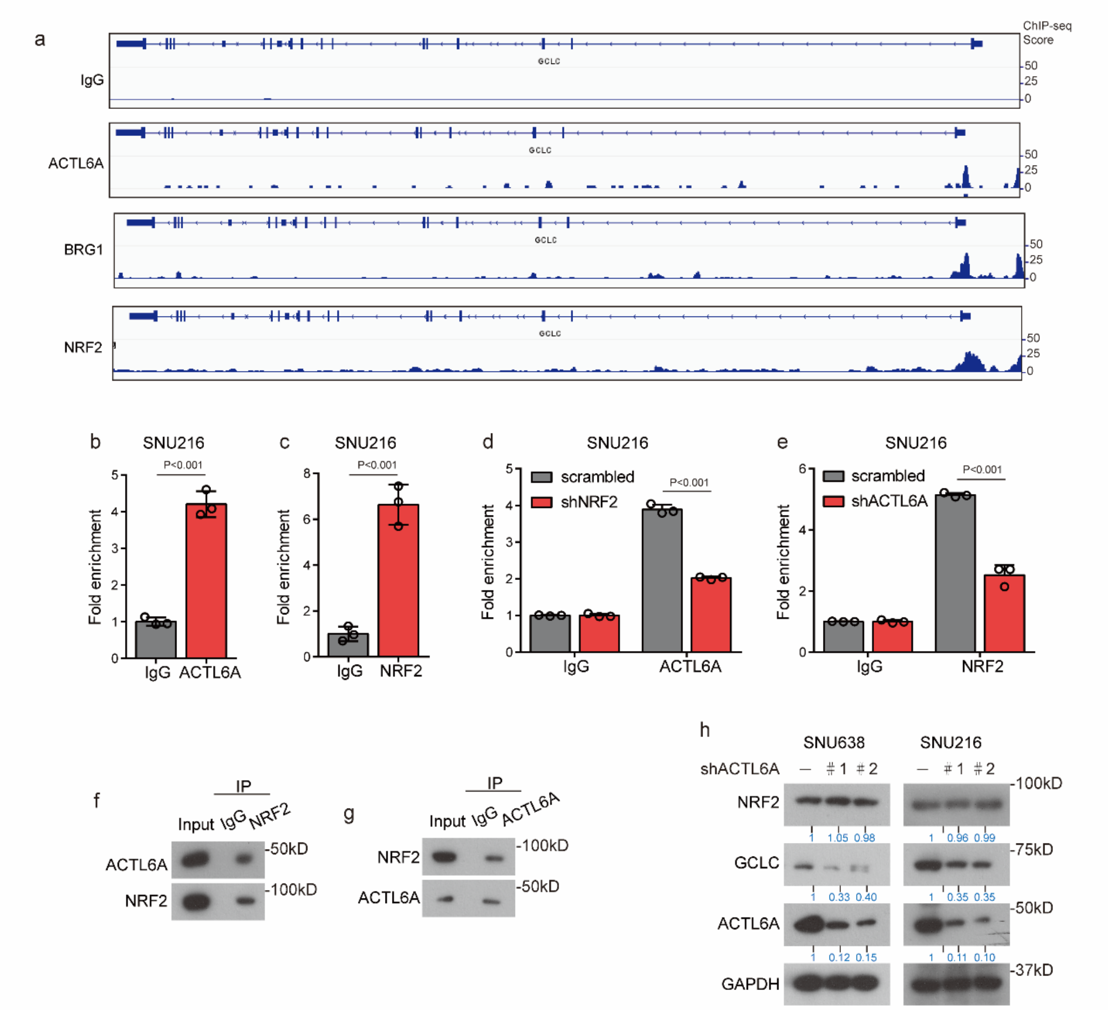

Supplementary figure 6 ACTL6A transcriptionally regulates GCLC dependent on NRF2. a. The entire genomic loci of GCLC in ACTL6A, BRG1 and NRF2 ChIP-seq results. IgG was used as a control. **b-c**. ChIP assay was performed in SNU216 cells using anti-ACTL6A (**b**) or anti-NRF2 (**c**) antibodies, followed by RT-qPCR with primers recognizing the predicting binding site of NRF2 in the transcriptional start of GCLC. The fold expression of ChIP-enriched mRNAs relative to the input was calculated, presented as the means ± SD, n=3 biologically independent experiments. IgG was used as a control. **d**. ChIP assay was performed in SNU216 cells treated with NRF2 shRNA or scrambled shRNA using anti-ACTL6A or anti-IgG antibodies, followed by RT-qPCR with primers recognizing the predicting binding site of NRF2 in the transcriptional start of GCLC. The fold expression of ChIP-enriched mRNAs relative to the input was calculated. The data are

presented as the means ± SD, n=3 biologically independent experiments. **e**. ChIP assay was performed in SNU216 cells treated with ACTL6A shRNA or scrambled shRNA using anti-NRF2 or anti-IgG antibodies, followed by RT-qPCR with primers recognizing the predicting binding site of NRF2 in the transcriptional start of GCLC. The fold expression of ChIP-enriched mRNAs relative to the input was calculated. The data are presented as the means ± SD, n=3 biologically independent experiments. **f**. Immunoblot analysis of the ACTL6A and NRF2 from anti-NRF2 immunoprecipitates (IP) obtained from SNU216 cells. Immunoglobulin G (IgG) serves as a control. **g**. Immunoblot analysis of the NRF2 and ACTL6A from anti-ACTL6A immunoprecipitates (IP) obtained from SNU216 cells. Immunoglobulin G (IgG) serves as a control. **h**. Immunoblot analysis of the NRF2, GCLC and ACTL6A protein levels in GC cells treated with ACTL6A shRNA or scrambled shRNA. *P* values were determined by unpaired two-tailed T test for panels **b-e**.

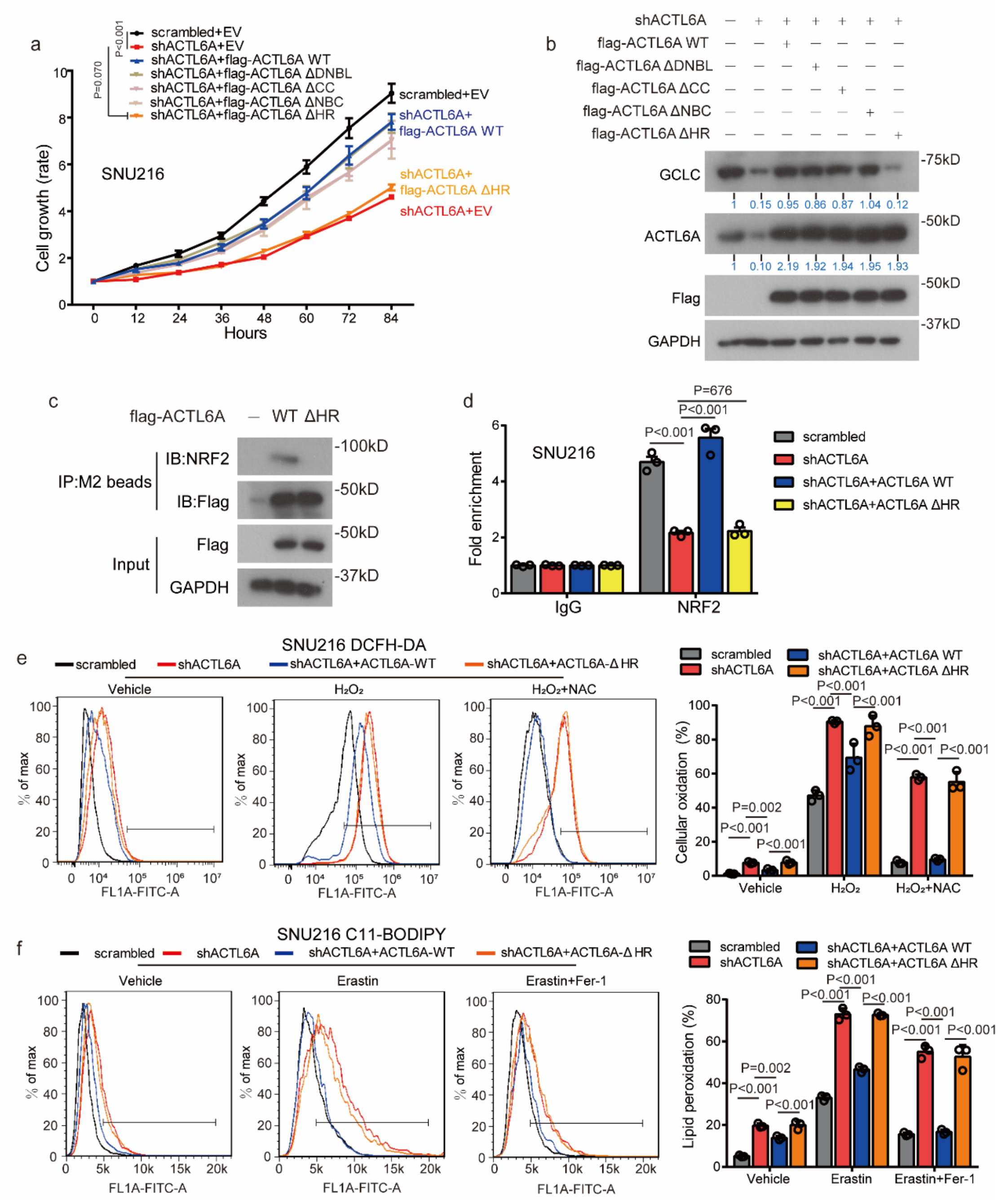

Supplementary figure 7 The HR domain of ACTL6A is essential in GCLC regulating and ferroptosis. a. Relative cell growth rate of SNU216 cells treated with ACTL6A shRNA or scrambled shRNA, and transfected with flag-ACTL6A-WT, constructs of domain deletion or pcDNA3.1 vector. The data are presented as the means ± SEM, n=3 biologically independent experiments. **b**. Immunoblotting analysis of the indicated proteins in cells from (**a**). **c**. Immunoblot analysis of the indicated proteins from M2 beads immunoprecipitates (IP) and whole cell lysates (input) obtained from SNU216

cells transfected with ACTL6A-WT, ACTL6A-ΔHR or pcDNA3.1 vector. **d**. ChIP assay was performed in SNU216 cells treated with ACTL6A shRNA or scrambled shRNA, and transfected with ACTL6A-WT, ACTL6A-ΔHR or pcDNA3.1 vector using anti-NRF2 or anti-IgG antibodies, followed by RT-qPCR with primers recognizing the predicting binding site of NRF2 in the transcriptional start of GCLC. The fold expression of ChIP-enriched mRNAs relative to the input was calculated. The data are presented as the means ± SD, n=3 biologically independent experiments. **e-f**. Relative DCFH-DA (**e**) and C11-BODIPY (**f**) fluorescence measured by flow cytometry of SNU216 cells with indicated treatment. The data are presented as the means ± SD, n=3 biologically independent experiments. *P* values were determined by two-way ANOVA followed by Tukey test for panel **a**, and unpaired two-tailed T test for panels **d-f**.

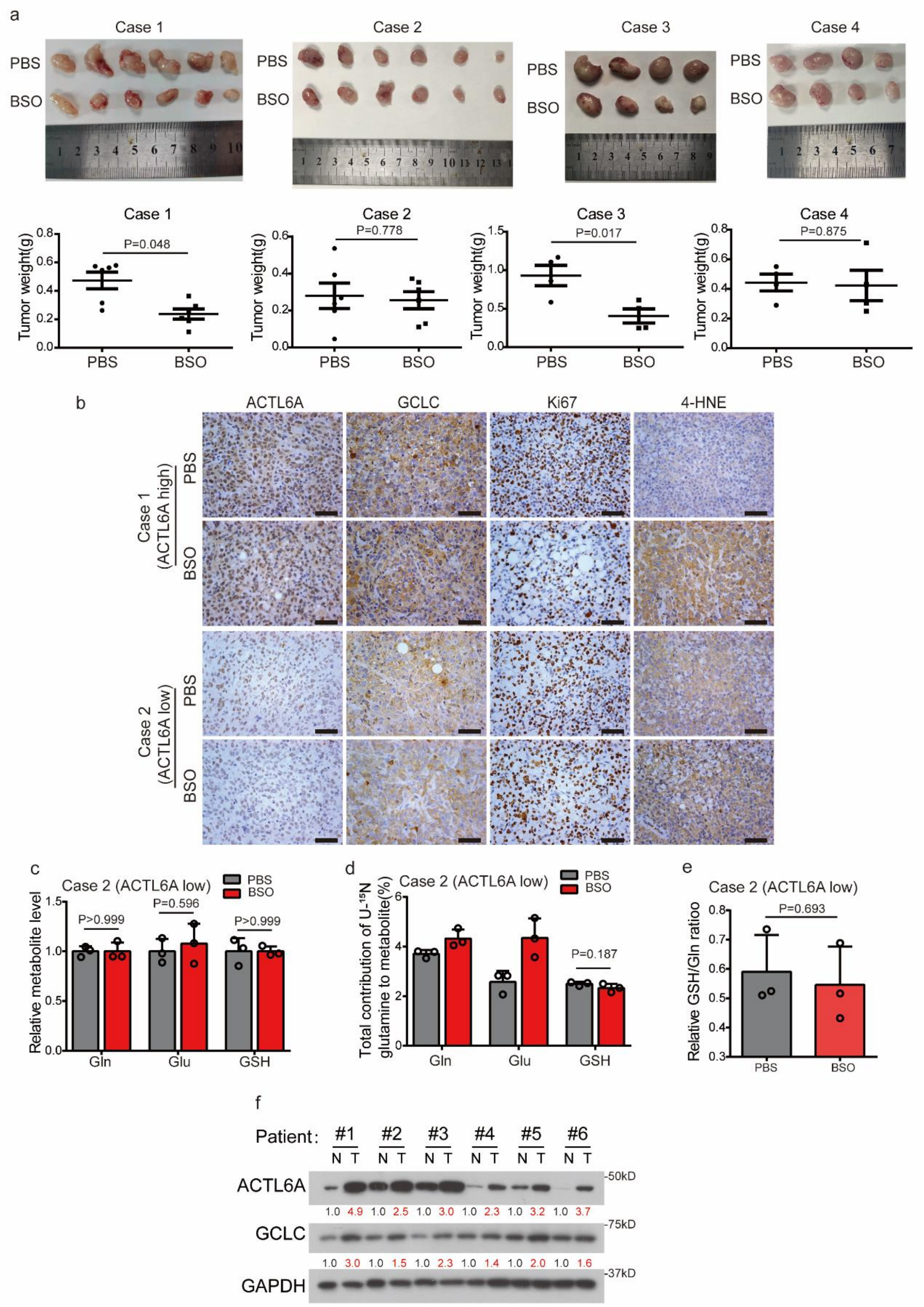

Supplementary figure 8 PDX tumors with ACTL6A high expression are more sensitive to BSO. a. Images and tumor weight of PDX tumors from 4 cases. Data of tumor weight are presented as the means ± SD, n=6 for each group in case 1 and 3, n=4 for each group in case 2 and 4. **b**. IHC analysis of

ACTL6A, GCLC, Ki-67 and 4-HNE staining in PDX tumor tissues generated from case 1 and case 2. Scale bars represent 50 μm. **c-e**. Relative intracellular pool levels of glutamine (gln), glutamate (glu) and GSH in case2 (ACTL6A low) PDX tumors (**c**). Incorporation of nitrogen atoms from [U-^{15}N] glutamine into glutamine (gln), glutamate (glu) and GSH (**d**). Relative GSH/Gln ratios (**e**). Data are presented as the means ± SD, n=3 for each group. **f**. Immunoblotting analysis of ACTL6A and GCLC protein levels from 6 paired samples of GC and normal tissue. *P* values were determined by unpaired two-tailed T test for panels **a, c-e**.

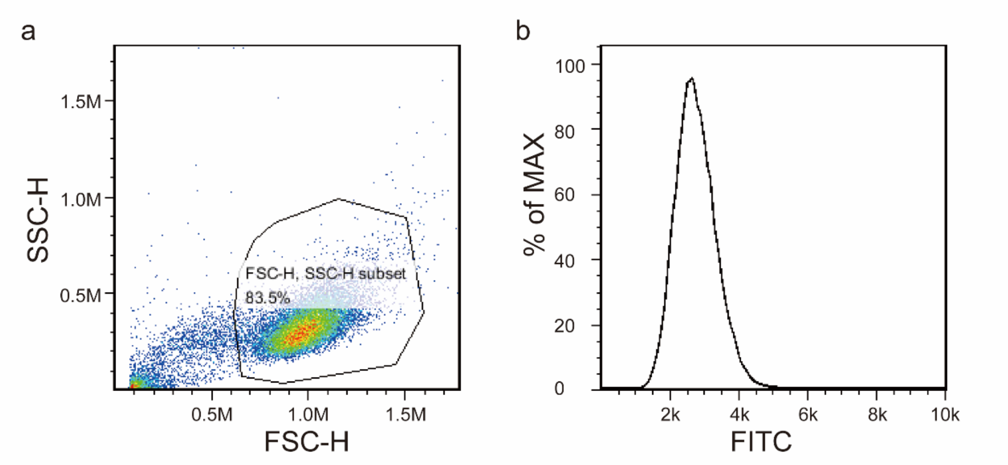

Supplementary figure 9 Gating strategy of Flow Cytometry in DCFH-DA and C11 BODIPY experiments. a. Live cells were gated from FSC/SSC plot. **b**. Cellular oxidation levels and lipid peroxidation levels of live cells were determined by FITC. The gating panel in (**b**) corresponds to all DCFH-DA and C11-BODIPY experiments.

Supplementary Table 1 Sequences for shRNAs targeting ACTL6A and NRF2

shRNA	Targeting sequences
shACTL6A(3'-UTR)	GCTTTCCTTGAAATGCACTTA
shACTL6A-1	CCCACCTACTACATAGATACT
shACTL6A-2	GGTACTTCAAGTGTCAGATTC
shNRF2	GCACCTTATATCTCGAAGTTT

Supplementary Table 2 Primers for RT-qPCR

Gene	Forward primer sequence	Reverse primer sequence
ACTL6A	TGGAGGCCATTTCACCTCTAA	TCTTTGCTCTAGTATTCCACGGT
GCLC	GGAGGAAACCAAGCGCCAT	CTTGACGGCGTGGTAGATGT
GCLM	TGTCTTGGAATGCACTGTATCTC	CCCAGTAAGGCTGTAAATGCTC
PTGS2	CTGGCGCTCAGCCATACAG	CGCACTTATACTGGTCAAATCCC
GPX2	GGTAGATTTCAATACGTTCCGGG	TGACAGTTCTCCTGATGTCCAAA
SLC7A11	TCTCCAAAGGAGGTTACCTGC	AGACTCCCCTCAGTAAAGTGAC
GSR	CACTTGCGTGAATGTTGGATG	TGGGATCACTCGTGAAGGCT
GPX4	GAGGCAAGACCGAAGTAAACTAC	CCGAACTGGTTACACGGGAA
GSS	GGGAGCCTCTTGCAGGATAAA	GAATGGGGCATAGCTCACCAC
GLS	AGGGTCTGTTACCTAGCTTGG	ACGTTCGCAATCCTGTAGATTT
SLC1A5	TCATGTGGTACGCCCCTGT	GCGGGCAAAGAGTAAACCCA

SLC3A2	TGAATGAGTTAGAGCCCGAGA	GTCTTCCGCCACCTTGATCTT
GAPDH	GGAGCGAGATCCCTCCAAAAT	GGCTGTTGTCATACTTCTCATGG

Supplementary Table 3 Primers of GCLC promoter for ChIP-qPCR

**Predicted binding site**	**Forward primer sequence**	**Reverse primer sequence**
GRCh38:6:53545016:53545243	TGTTCACCTCATATGGCTGTAGT	TGGAAAAGATCAAGGAGGCTGA
