# Supplementary material for: ACTL6A protects gastric cancer cells against ferroptosis through induction of glutathione synthesis
Source: Nat Commun. 2023 Jul 13;14:4193. doi: 10.1038/s41467-023-39901-8 (PMC10345109; doi:10.1038/s41467-023-39901-8)
Supplement: Supplementary file 3 — Reporting Summary [file 41467_2023_39901_MOESM3_ESM.pdf]

nature portfolio

Corresponding author(s): Lekun Fang

Last updated by author(s): Mar 13, 2023

Reporting Summary

Nature Portfolio wishes to improve the reproducibility of the work that we publish. This form provides structure for consistency and transparency in reporting. For further information on Nature Portfolio policies, see our Editorial Policies and the Editorial Policy Checklist.

Statistics

For all statistical analyses, confirm that the following items are present in the figure legend, table legend, main text, or Methods section.

n/a	Confirmed	
☐	☒	The exact sample size (*n*) for each experimental group/condition, given as a discrete number and unit of measurement
☐	☒	A statement on whether measurements were taken from distinct samples or whether the same sample was measured repeatedly
☐	☒	The statistical test(s) used AND whether they are one- or two-sided<br>*Only common tests should be described solely by name; describe more complex techniques in the Methods section.*
☒	☐	A description of all covariates tested
☒	☐	A description of any assumptions or corrections, such as tests of normality and adjustment for multiple comparisons
☐	☒	A full description of the statistical parameters including central tendency (e.g. means) or other basic estimates (e.g. regression coefficient) AND variation (e.g. standard deviation) or associated estimates of uncertainty (e.g. confidence intervals)
☐	☒	For null hypothesis testing, the test statistic (e.g. *F*, *t*, *r*) with confidence intervals, effect sizes, degrees of freedom and *P* value noted<br>*Give P values as exact values whenever suitable.*
☒	☐	For Bayesian analysis, information on the choice of priors and Markov chain Monte Carlo settings
☒	☐	For hierarchical and complex designs, identification of the appropriate level for tests and full reporting of outcomes
☐	☒	Estimates of effect sizes (e.g. Cohen's *d*, Pearson's *r*), indicating how they were calculated

Our web collection on statistics for biologists contains articles on many of the points above.

Software and code

Policy information about availability of computer code

Data collection	RT-qPCR data were collected by Design and analysis 2.6.0.<br>Cell growth curves were collected by incucyte ZOOM 2016B.<br>Cell viability data were collected by PerkinElmer VICTOR Nivo.<br>Florescence pictures were collected by OLYMPUS cellSens Standard 2.2.<br>IHC pictures were collected by ImageViewerG.Ink.
Data analysis	Western blot quantification and fluorescence intensity were analyzed by ImageJ-win64 (v1.48).<br>All statistical data analyses were performed using Graphpad Prism 7 software and IBM SPSS Statistics 25.<br>Flow Cytometry results were analyzed by FlowJo 7.6.1.<br>Gene Set Enrichment Analysis (GSEA) was performed with GSEA_4.1.0.<br>ChIP-seq experiments was analyzed by BWA (v0.7.17-r1188), Fastp (v0.23.0), FastQC (v0.11.9), Deeptools2 (v3.5.1), ATACseqQC (v1.16.0), MACS2 (v2), ChIPseeker (v1.28.3), HOMER (v2), DiffBind (v3.2.7) and clusterProfiler (v3.1).

For manuscripts utilizing custom algorithms or software that are central to the research but not yet described in published literature, software must be made available to editors and reviewers. We strongly encourage code deposition in a community repository (e.g. GitHub). See the Nature Portfolio guidelines for submitting code & software for further information.

Data

Policy information about availability of data

All manuscripts must include a data availability statement. This statement should provide the following information, where applicable:

- Accession codes, unique identifiers, or web links for publicly available datasets
- A description of any restrictions on data availability
- For clinical datasets or third party data, please ensure that the statement adheres to our policy

The RNA array data generated in this study have been deposited in the Gene Expression Omnibus (GEO) database under accession code GSE203657. The anti-ACTL6A, anti-BRG1 and anti-NRF2 ChIP-seq data generated in this study have been deposited in the Gene Expression Omnibus (GEO) database under accession code GSE216350. Publicly available datasets reported in this paper are from the GEO databases (GSE13911, GSE27342 and GSE13861) and The Cancer Genome Atlas. Source data are provided with this paper.

Human research participants

Policy information about studies involving human research participants and Sex and Gender in Research.

Reporting on sex and gender	Sex or gender was not considered in the study design.
Population characteristics	70.1% (129/184) of them were male, and 29.9% (55/184) were female. 34.8% (64/184) of them were under 59 years old, and 65.2% (120/184) of them were no less than 59 years old. The detailed patient characteristics were listed in Supplementary Table 4.
Recruitment	We obtained paraffin-embedded samples of primary gastric adenocarcinomas (prepared as Tissue Microarray, TMA) from the Department of Surgery at the Sixth Affiliated Hospital of Sun Yat-sen University.
Ethics oversight	All samples were collected with the patients' written informed consent and approval from the Sixth Affiliated Hospital of Sun Yat-sen University Review Board (ethics code: 2021ZSLYEC-100).

Note that full information on the approval of the study protocol must also be provided in the manuscript.

Field-specific reporting

Please select the one below that is the best fit for your research. If you are not sure, read the appropriate sections before making your selection.

[x] Life sciences [ ] Behavioural & social sciences [ ] Ecological, evolutionary & environmental sciences

For a reference copy of the document with all sections, see nature.com/documents/nr-reporting-summary-flat.pdf

Life sciences study design

All studies must disclose on these points even when the disclosure is negative.

Sample size	For in vitro study, all the experiments were repeated at least 3 independent experiments that are commonly used to estimate the effect of the treatment. For animal study, sample size was determined to be adequate based on the magnitude and consistency of measurable differences between groups.
Data exclusions	No data were excluded form analyses.
Replication	The reproducibility for each analysis confirmed at least two times
Randomization	For in vitro study using cell lines and PDOs, samples were analyzed equally with no sub-sampling and thus there was no requirement for randomization. In in vivo experiments, mice of 4-8 weeks of age were used, which were randomly divided to different treatment groups after cell injections.
Blinding	Randomization and single blinding were performed when measuring tumor volume (volume = 0.5 × length × width × width) and weight.

Reporting for specific materials, systems and methods

We require information from authors about some types of materials, experimental systems and methods used in many studies. Here, indicate whether each material, system or method listed is relevant to your study. If you are not sure if a list item applies to your research, read the appropriate section before selecting a response.

Materials & experimental systems

n/a	Involved in the study	
☐	☒	Antibodies
☐	☒	Eukaryotic cell lines
☒	☐	Palaeontology and archaeology
☐	☒	Animals and other organisms
☒	☐	Clinical data
☒	☐	Dual use research of concern

Methods

n/a	Involved in the study	
☐	☒	ChIP-seq
☐	☒	Flow cytometry
☒	☐	MRI-based neuroimaging

Antibodies

Antibodies used

Antibodies specific for ACTL6A (Abcam, ab3882), GCLC (Abcam, ab190685), NRF2 (Cell Signaling Technology, 12721s), Ki67 (1:400, Cell Signaling Technology, 9449), 4-HNE (1:4000, Abcam, ab46545), flag tag (Sigma, F1804), BRG1 (Proteintech, 21634-1-AP) and GAPDH (Proteintech, 10494-1-AP). IgG control antibody （normal rabbit IgG ： Millipore cat.no. 12-370, normal mouse IgG ： Millipore cat.no. 12-371） and secondary antibody (Anti-Rabbit IgG antibody, Goat monoclonal: Millipore AP132).

Validation

Validation statement of each antibody was noted on the manufacturer website:
1.anti-ACTL6A: https://www.abcam.cn/products/primary-antibodies/baf53a-antibody-chip-grade-ab3882.html
2.anti-GCLC: https://www.abcam.cn/products/primary-antibodies/gclc-antibody-ep13475-ab190685.html
3.anti-NRF2: https://www.cellsignal.com/products/primary-antibodies/nrf2-d1z9c-xp-rabbit-mab/12721
4.anti-Ki67: https://www.cellsignal.com/products/primary-antibodies/ki-67-8d5-mouse-mab/9449
5.anti-4-HNE: https://www.abcam.cn/products/primary-antibodies/4-hydroxynonenal-antibody-ab46545.html
6.anti-FLAG: https://www.sigmaaldrich.cn/CN/en/product/sigma/f1804
7.anti-BRG1: https://www.ptglab.co.jp/Products/SMARCA4-Antibody-21634-1-AP.htm
8.anti-GAPDH: https://www.ptglab.co.jp/products/GAPDH-Antibody-10494-1-AP.htm

Eukaryotic cell lines

Policy information about cell lines and Sex and Gender in Research

Cell line source(s)

SNU638, SNU216 and SNU668 cells were obtained from KCLB (Korean Cell Line Bank), and HEK 293T cells were obtained from ATCC (American Type Culture Collection)

Authentication

The cell were authenticated by STR profilling

Mycoplasma contamination

All cell lines were tested negative for mycoplasma contamination.

Commonly misidentified lines (See ICLAC register)

No commonly misidentified cell lines were used.

Animals and other research organisms

Policy information about studies involving animals; ARRIVE guidelines recommended for reporting animal research, and Sex and Gender in Research

Laboratory animals

Mice were purchased from GemPharmatech Biotechnology Corporation. 4-8 weeks of BALB/ c-nu/nu mice and NCG mice were used in this study.

Wild animals

No wild animals were used in this study.

Reporting on sex

Sex was not considered in the study design.

Field-collected samples

No field-collected samples were used in this study.

Ethics oversight

Animal experiments were approved by the Institutional Animal Care and Use Committee of The Sixth Affiliated Hospital of Sun Yat-sen University (IACUC-2021011501).

Note that full information on the approval of the study protocol must also be provided in the manuscript.

ChIP-seq

Data deposition

☒ Confirm that both raw and final processed data have been deposited in a public database such as GEO.

☒ Confirm that you have deposited or provided access to graph files (e.g. BED files) for the called peaks.

Data access links
May remain private before publication.

GSE216350

Files in database submission

anti-ACTL6A: SNU638-ACTL6A_1.fq; SNU638-ACTL6A_2.fq; SNU638-ACTL6A.fpkm.bigwig; SNU638-ACTL6A.peak.bed;
anti-BRG1: SNU638-BRG1_1.fq; SNU638-BRG1_2.fq; SNU638-BRG1.fpkm.bigwig; SNU638-BRG1.peak.bed.
anti-NRF2: SNU638-NRF2_1.fq; SNU638-NRF2_2.fq; SNU638-NRF2.fpkm.bigwig; SNU638-NRF2.peak.bed.
IgG: SNU638-IgG_1.fq; SNU638-IgG_2.fq; SNU638-IgG.fpkm.bigwig; SNU638-IgG.peak.bed.

Genome browser session
(e.g. UCSC)

N/A

Methodology

Replicates

These experiments aimed to explore the common binding site of ACTL6A, BRG1 and NRF2. We did not compare after any treatment. Therefore, no replicates was used.

Sequencing depth

anti-ACTL6A: 43,373,174 raw reads, 35,902,116 clean reads.
anti-BRG1: 52,726,700 raw reads, 44,678,078 clean reads.
anti-NRF2: 57,982,332 raw reads, 57,524,666 clean reads.
IgG: 50,777,508 raw reads, 50,308,374 clean reads.
All samples have sequences of a single length (150bp).
They were paired-end 150bp.

Antibodies

A primary antibody (anti-ACTL6A, Abcam, ab3882; anti-BRG1, Proteintech, 21634-1-AP; anti-NRF2, Cell Signaling Technology, 12721s) or IgG control antibody （normal rabbit IgG：Millipore cat.no. 12-370， normal mouse IgG：Millipore cat.no. 12-371）

Peak calling parameters

The bam file generated by the unique mapped reads as an input file, using macs2 software for callpeak with cutoff qvalue < 0.05.

Data quality

Raw data (raw reads) of fastq format were firstly processed through in-house perl scripts. In this step, clean data (clean reads) were obtained by removing reads containing adapter, reads containing ploy-N and low quality reads from raw data. At the same time, Q20, Q30 and GC content the clean data were calculated. All the downstream analyses were based on the clean data with high quality.

Software

BWA (v0.7.17-r1188),
Fastp (v0.23.0),
FastQC (v0.11.9),
Deeptools2 (v3.5.1),
ATACseqQC (v1.16.0),
MACS2 (v2),
ChIPseeker (v1.28.3),
HOMER (v2),
DiffBind (v3.2.7),
clusterProfiler (v3.1)

Flow Cytometry

Plots

Confirm that:

- [x] The axis labels state the marker and fluorochrome used (e.g. CD4-FITC).
- [x] The axis scales are clearly visible. Include numbers along axes only for bottom left plot of group (a 'group' is an analysis of identical markers).
- [x] All plots are contour plots with outliers or pseudocolor plots.
- [x] A numerical value for number of cells or percentage (with statistics) is provided.

Methodology

Sample preparation

DCFH-DA or C11-BODIPY staining for 30 min

Instrument

Beckman CytoFLEX SRT

Software

Flowjo 7.6

Cell population abundance

20,000 cell were recorded for each sample

Gating strategy

Live cells were gated from FSC/SSC plot. Then cellular oxidation levels and lipid peroxidation levels of live cells were determined by FITC.

- [x] Tick this box to confirm that a figure exemplifying the gating strategy is provided in the Supplementary Information.
