# Supplementary material for: ACTL6A protects gastric cancer cells against ferroptosis through induction of glutathione synthesis
Source: Nat Commun. 2023 Jul 13;14:4193. doi: 10.1038/s41467-023-39901-8 (PMC10345109; doi:10.1038/s41467-023-39901-8)
Supplement: Supplementary file 4 — Description of Additional Supplementary Files [file 41467_2023_39901_MOESM4_ESM.pdf]

Description of Additional Supplementary Files

Supplementary Data 1
Description: Correlation between expression of ACTL6A-GCLC axis and clinicopathological features of gastric cancer patients
